# Association of *Fusobacterium nucleatum* in human saliva with periodontal status and composition of the salivary microbiome including periodontopathogens

**DOI:** 10.1128/spectrum.00855-24

**Published:** 2024-10-22

**Authors:** Takanori Akase, Junya Inubushi, Yoshiko Hayashi-Okada, Yasumitsu Shimizu

**Affiliations:** 1 Oral Care R&D Department, Sunstar Inc., Osaka, Japan; University of Manitoba, Winnipeg, Manitoba, Canada

**Keywords:** *Fusobacterium*, 16S rRNA, red complex, oral microbiome

## Abstract

*Fusobacterium nucleatum* promotes dental biofilm formation, increases the prevalence of periodontal disease, and is associated with systemic diseases such as colorectal cancer. However, differences in the composition of salivary microbiome among groups based on the abundance of *F. nucleatum* are unclear. Therefore, we analyzed the difference in salivary microbiome among groups based on the abundance of *F. nucleatum* in saliva samples collected from 611 subjects in Japan. Salivary DNA was extracted, and the oral microbiome was analyzed using next-generation sequencing of 16S rRNA. The relationship between *F. nucleatum* and the community periodontal index was evaluated to examine effects on periodontal status, and α- and β-diversity were analyzed in four groups based on quantiles of relative abundance of *F. nucleatum*. Spearman rank correlation tests were used to examine relationships between the relative abundance of *F. nucleatum* and oral bacteria. Subjects with the highest quantiles of *F. nucleatum* had a higher prevalence of periodontitis compared with those with the lowest quantile. β-Diversity also differed among these four groups. *F. nucleatum* showed significant correlations with several periodontopathogens, including the red complex, *Prevotella intermedia*, *Filifactor alocis,* and *Fretibacterium* ssp. These results suggest that the relative abundance of *F. nucleatum* in saliva is associated with periodontal status and the composition of the salivary microbiome, including the red complex and periodontopathogens.

**IMPORTANCE:**

We characterized the composition of the saliva microbiome in groups based on the abundance of *Fusobacterium nucleatum*. There is a lot of periodontitis in subjects with a high abundance of *F. nucleatum*. The diversity of the saliva microbiome was different among groups based on the abundance of *F. nucleatum*. The abundance of *F. nucleatum* correlated with various periodontopathogens including red complex. These results support the influence of the abundance of *F. nucleatum* in saliva on periodontal status and the composition of the salivary microbiome, including the red complex and periodontopathogens.

## INTRODUCTION

Periodontitis involves inflammation of periodontal tissue caused by bacterial infection and is clinically characterized by periodontal pocket formation and alveolar bone resorption (1). The oral cavity harbors a complex and diverse oral microbiome comprising more than 700 different predominant oral microbes (2). Various oral microbes are also associated with periodontal disease (3). For example, *Porphyromonas gingivalis, Treponema denticola,* and *Tannerella forsythia* are classically known as subgingival bacteria and members of the Socransky red complex because of their co-aggregation characteristics and strong association with periodontal disease (4, 5). The high relative abundance of periodontopathogens in the oral microbiome changes the composition of the oral microbiome, causes dysbiosis of the oral microbiome, and leads to periodontal disease. Hence, identifying the oral microbes associated with the composition of the oral microbiome is necessary to prevent periodontal disease; however, they are currently unclear.

*Fusobacterium nucleatum* is an oral microbe that has been detected at various sites in the oral cavity. *F. nucleatum* is a member of the Socransky orange complex, which plays a role in the development and progression of periodontal disease (4). Recently, *F. nucleatum* has also been associated with colorectal cancer (6) and has important roles in the development of oral and systemic disease. *F. nucleatum* colonizes subgingival plaque and bridges between early and late colonizers in the development of dental biofilm (7), including that by periodontopathogens. Thus, *F. nucleatum* can co-aggregate with the red complex to enhance biofilm formation (8–10). Several studies have reported a strong correlation between the microbial profile of the salivary microbiome and subgingival plaque (11–14).

The above findings suggest that the relative abundance of *F. nucleatum* in saliva may correlate with various effects of periodontopathogens and may affect the composition of the salivary microbiome. A recent study found that the relative abundance of *F. nucleatum* and red complex in the salivary microbiome predicts the periodontal status with high sensitivity (15). However, few data are available on the relationship between the composition of the saliva microbiome and the relative abundance of *F. nucleatum*. Therefore, in the current study, we performed a comprehensive analysis of the saliva microbiome by sequencing the V3-V4 region of 16S rRNA, with a particular focus on the association of *F. nucleatum* with the community periodontal index (CPI), which is a clinical marker for periodontal status. CPI have been utilized as periodontal status in some papers (16–18). We also examined the difference in diversity among groups classified by the relative abundance of *F. nucleatum* to assess the correlation between *F. nucleatum* and oral microbes in saliva. Collectively, the results show the influence of the abundance of *F. nucleatum* in saliva on periodontal status and the composition of the salivary microbiome, including the red complex and periodontopathogens.

## MATERIALS AND METHODS

### Community periodontal index

The periodontal status of each subject was evaluated using the CPI. CPI was assessed using a modified version of the World Health Organization (WHO) Oral Health Surveys—Basic Methods guidelines. CPI measurements were performed with a periodontal probe (WHO probe). Each sextant was assigned a code, and three indicators (bleeding, calculus, and pocket depth) were used to assess the CPI: healthy gingiva (CPI = 0); gingival bleeding (CPI = 1); calculus (CPI = 2); and periodontal pockets 4–5 mm (CPI = 3) and ≥6 mm (CPI = 4) in each sextant. All teeth were examined to screen for periodontal status. The CPI score of each subject is the highest score among all teeth.

### Sample collection

Saliva was collected using OMNIgene (OM-501, DNA Genotek) by the subject. All subjects were instructed to refrain from eating and drinking for at least 30 min and from using oral hygiene products for at least 2 hours prior to collection. The collected saliva was stored at room temperature until DNA extraction.

### DNA extraction

DNA was extracted from saliva samples using a Qiamp DNA Mini Kit (Qiagen Japan). Briefly, 400 µL of samples containing saliva was incubated at 56°C for 1 hour and then centrifuged at 12,000 rpm for 5 min. The supernatant was combined in a tube with 400 µL of lysis buffer and incubated at 56°C. A total of 400 µL of ethanol was added, and the mixture was applied to a spin column and centrifuged at 6,000 × *g* for 1 min. The precipitate was combined with 200 µL of 1,000 U achromopeptidase (Wako, Japan) and incubated at 56°C. After the addition of 20 µL of proteinase K and 180 µL of lysis buffer, the mixture was further incubated at 56°C. It was then combined with 200 µL of ethanol, applied to a spin column, and centrifuged at 6,000 × *g* for 1 min. The spin column was washed twice with wash buffer. DNA was eluted from the spin column using distilled water (UltraPure DNase/RNase-Free Distilled water, Thermo Fisher). All batches of samples were performed with DNA extraction negative controls and positive controls from saliva samples.

### 16S rRNA amplicon sequencing

Metagenomic amplification and 16S rRNA amplicon sequencing of the extracted DNA were performed according to illumina's method. Briefly, metagenomic DNA was amplified using the 16S V3 (341F) forward and V4 (805R) reverse primer pairs with added Illumina adapter overhang nucleotide sequences. Amplicon PCR was completed with 10.5 µL of genomic DNA, 1 µL of amplicon PCR forward primer (5 µM), 1 µL of amplicon PCR reverse primer (5 µM), and 12.5 µL of 2× KAPA HiFi HotStart Ready Mix (Kapa Biosystems) at 95°C for initial denaturation for 3 min, followed by 25 cycles of 95°C for 30 s, 62.3°C for 30 s, and 72°C for 30 s, and final extension at 72°C for 5 min. Reactions were cleaned with Agencourt AMPure XP beads (Beckman Coulter Genomics, South Plainfield, NJ). Library generation was performed using 5 µL of amplicon PCR product DNA, 5 µL of Illumina Nextera XT Index Primer 1 (N7xx), 5 µL of Nextera XTIndex Primer 2 (S5xx), 25 µL of 2× KAPA HiFi HotStart Ready Mix, and 10 µL of PCR-grade water (UltraClean MO BIO Laboratories, Inc.), with thermocycling at 95°C for 3 min, followed by eight cycles at 95°C for 30 s, 55°C for 30 s, and 72°C for 30 s, and final extension at 72°C for 5 min. 16S metagenomic libraries were purified with Agencourt AMPure XP beads and quantified with Quant-iT PicoGreen. Nextera index primer sets (A, B, and C) were rotated for each batch to reduce sequence carryover between MiSeq runs. Library quality control was performed with a Fragment Analyzer (Advanced Analytical Technologies, Inc., Ankeny, IA) to ascertain average size distribution. The pool of normalized libraries was then quantified with the NEBNext Library Quant Kit (New England Biolabs, Inc., Ipswich, MA), denatured with NaOH, and diluted to a final concentration of 10 pM with a 20% PhiX (Illumina, Inc., San Diego, CA). 2 × 300 bp paired-end sequencing was performed in the Illumina MiSeq System (Illumina, Inc.) by multiplexing 96 samples per sequencing run with the MiSeq Reagent Kit.

### Bioinformatics and statistical analysis

Joining of Illumina paired-end reads was completed using Paired-End reAd mergeR (PEAR ver. 0.9.6). The percentage of successfully joined paired ends was defined as the “merge rate”; paired-end reads that could not be joined were removed from downstream analyses. Sequence quality filtering was done with the Fastx-Toolkit (V.0.013) to isolate reads with 90% of bases having a score higher than Q30, which defined the “pass rate”; reads not meeting this criterion were removed. Primer sequences were trimmed based on the length of the forward and reverse sequencing primers. Following quality filtering, reads were deduplicated by recording the number and type of identical sequences to reduce the downstream processing time. Taxonomy annotation was performed with BLAST at a 97% similarity for species-level assignment approximation against bacterial sequences from HOMD ver. 11.0. Input query reads were given the same taxonomic label as the best hit in the reference sequence collection, defining the “hit count”; reads with no hits were excluded from downstream analyses. Sequences with the same labels were clustered into one OTU (Operational Taxonomic Unit), and the raw OTU table was constructed by combining absolute sequence abundancies from the deduplication step, generated taxonomy annotations, and manually generated metadata. The preprocessing sequence analysis step required a merge rate ≥90%, pass rate ≥60%, and hit count per sample ≥3,000. The relative abundance of *F. nucleatum* was defined as the total abundance of *F. nucleatum* ssp. *animalis*, *nucleatum*, *plantarum,* and *vincentii*. The relative abundance of red complex was defined as the total abundance of *P. gingivalis*, *T. denticola,* and *T. forsythia*.

Subjects were categorized into four groups based on quantiles of relative abundance of *F. nucleatum* and into four different groups based on quantiles of relative abundance of red complex. A comparison of the number of subjects with a different CPI among the abundance groups was performed by the Fisher exact test. The α- and β-diversity were calculated using R (ver. 4.1.0) package “vegan” (ver. 2.5-7). Box plots and plots were made in R (ver. 4.1.0) package “ggplot2” (ver. 3.3.3). α-Diversity was calculated in terms of species count and Shannon, Chao1, and Simpson indexes between groups using a Kruskal–Wallis pairwise test. A comparison of differences in all groups based on the relative abundance of total *F. nucleatum* in β-diversity was calculated using permutational multivariate analysis of variance (PERMANOVA) with a permutation of 999. A Spearman rank test was performed to examine the correlation of the relative abundance of *F. nucleatum* with other salivary species. For these analyses, an adjusted *P*-value of <0.05 was considered significant with a Benjamini–Hochberg correction.

## RESULTS

### Subjects

The characteristics of the 611 subjects (435 men and 176 women, 20–75 years old) are shown in Table 1. A total of 611 saliva samples were analyzed by 16S rRNA gene amplicon analysis, and high-quality reads were obtained that allowed the determination of the relative abundance of bacteria in the saliva.

**TABLE 1:** Demographics of the subjects

Parameter	Number of subjects (%)	
Gender	Men (*n* = 435)	Women (*n* = 176)
Age group		
20–29	30 (6.9%)	33 (18.8%)
30–39	65 (14.9%)	49 (27.8%)
40–49	151 (34.7%)	63 (35.8%)
50–59	147 (33.8%)	26 (14.8%)
≥60	42 (9.7%)	5 (2.8%)
CPI		
0	67 (15.4%)	67 (38.1%)
1, 2	189 (43.4%)	70 (39.8%)
3, 4	179 (41.1%)	39 (22.2%)

### Association of relative abundance of *F. nucleatum* with CPI and age

The number of subjects in each quantile in each gender, age, and CPI category is shown in Table 2. To confirm that the distribution of subjects with CPI is different between the highest quantile group and the lowest quantile group for the total *F. nucleatum*, we analyzed the differences in the ratio of subjects using the Fisher exact test. There were significantly higher rates of CPI 3 or 4 in the highest quantile for the total *F. nucleatum* compared to the lowest quantile (Table 2, *P* < 0.001). There were also higher rates of CPI 3 or 4 in the highest quantile for *F. nucleatum* ssp. *animalis* (*P* < 0.001), *nucleatum* (*P* < 0.005), *polymorphum* (*P* < 0.005), and *vincentii* (*P* < 0.001) compared to the lowest quantile (Table S1). There were significantly higher rates for the 50–59-year-old age group in the highest quantile for the total *F. nucleatum* compared to the lowest quantile (Table 2, *P* < 0.001).

**TABLE 2:** Distribution of CPI in groups classified based on the relative abundance of *F. nucleatum^a^*

Parameter	Number of subjects (%)				*P*-value
	Quantile1	Quantile2	Quantile3	Quantile4	Q1 vs Q4
Gender					
Man	97 (63.4%)	109 (71.7%)	117 (76.5%)	112 (73.2%)	*P* = 0.085
Woman	56 (36.6%)	43 (28.3%)	36 (23.5%)	41 (26.8%)	
Age group					
20–29	22 (14.4%)	17 (11.2%)	13 (8.5%)	11 (7.2%)	*P* < 0.001
30–39	41 (26.8%)	29 (19.1%)	22 (14.4%)	22 (14.4%)	
40–49	53 (34.6%)	53 (34.9%)	56 (36.6%)	52 (34.0%)	
50–59	26 (17.0%)	44 (28.9%)	46 (30.1%)	57 (37.3%)	
≥60	11 (7.2%)	9 (5.9%)	16 (10.5%)	11 (7.2%)	
CPI					
0	59 (38.6%)	30 (19.7%)	31 (20.3%)	14 (9.2%)	*P* < 0.001
1, 2	60 (39.2%)	73 (48.0%)	69 (45.1%)	57 (37.3%)	
3, 4	34 (22.2%)	49 (32.2%)	53 (34.6%)	82 (53.6%)	

^*a*^ Comparison of the distribution of gender, age, and CPI between the lowest quantile group (Q1) and the highest quantile group (Q4) was performed by the Fisher exact test.

### Diversity analysis

**Fig 1:**
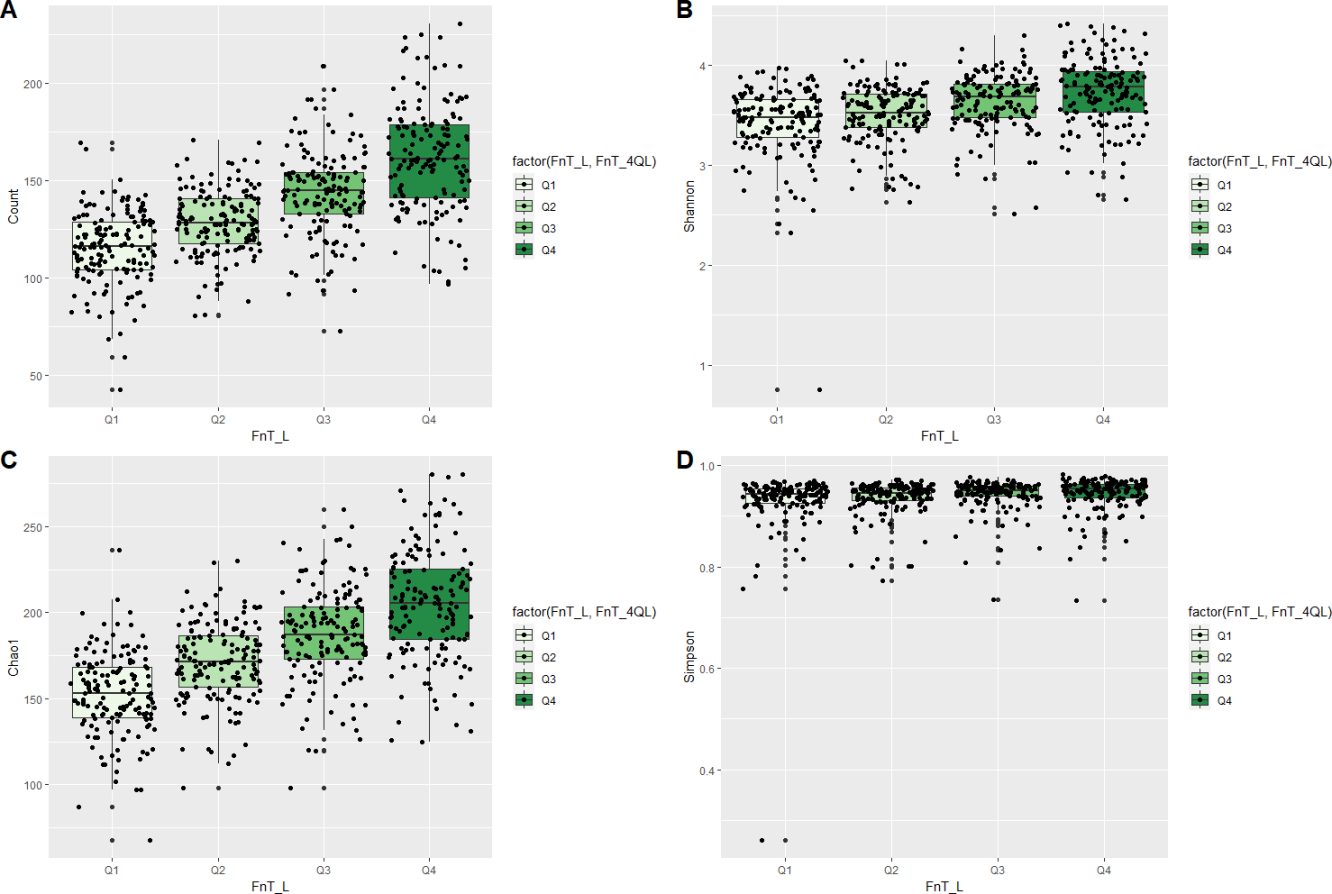
α-Diversity of the salivary microbiome. Box plots show the number of observed species (**A**), Shannon index (**B**), Chao1 index (**C**), and Simpson index (**D**) of each sampling method with the number of sequences rarefied to 3,000 reads per sample. Subjects were categorized into four groups based on quantiles of relative abundance of *F. nucleatum*. Q1, Quantile1; Q2, Quantile2; Q3, Quantile3; Q4, Quantile4. Comparison of the value of α-diversity between the lowest quantile group (**Q1**) and the highest quantile group (**Q4**) was performed by the Wilcoxon rank sum test.

**Fig 2:**
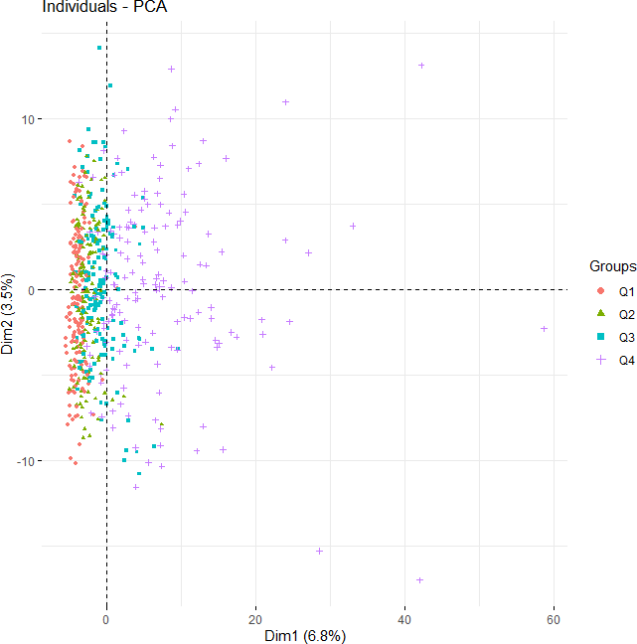
β-Diversity of the salivary microbiome classified based on relative abundance of *F. nucleatum*. Principal component analysis of salivary microbiota is shown. Each point represents an individual sample. Subjects were categorized into four groups based on quantiles of relative abundance of *F. nucleatum*. Q1, Quantile1; Q2, Quantile2; Q3, Quantile3; Q4, Quantile4. Comparison of difference among all groups in β-diversity was performed by PERMANOVA with a permutation of 999.

An evaluation of the effects of the relative abundance of *F. nucleatum* on α-diversity (Fig. 1) showed that species counts and the Shannon, Chao1, and Simpson indexes were significantly higher in the highest quantile than in the lowest quantile (*P* < 0.05). The relative abundance of *F. nucleatum* was significantly correlated with the species count and Shannon index. β-Diversity was analyzed to assess the influence of *F. nucleatum* on the composition of the salivary microbiome, using principal component analysis classified based on the abundance of *F. nucleatum* (Fig. 2). β-Diversity differed significantly among the four quantiles (*P* = 0.001). It also showed a significant difference among four quantiles based on the relative abundance of each *F. nucleatum* ssp. *animalis* (*P* = 0.002), *nucleatum* (*P* = 0.044), *polymorphum* (*P* = 0.001), and *vincentii* (*P* = 0.001) (Fig. S1).

### Bacteria correlating with *F. nucleatum*

**Fig 3:**
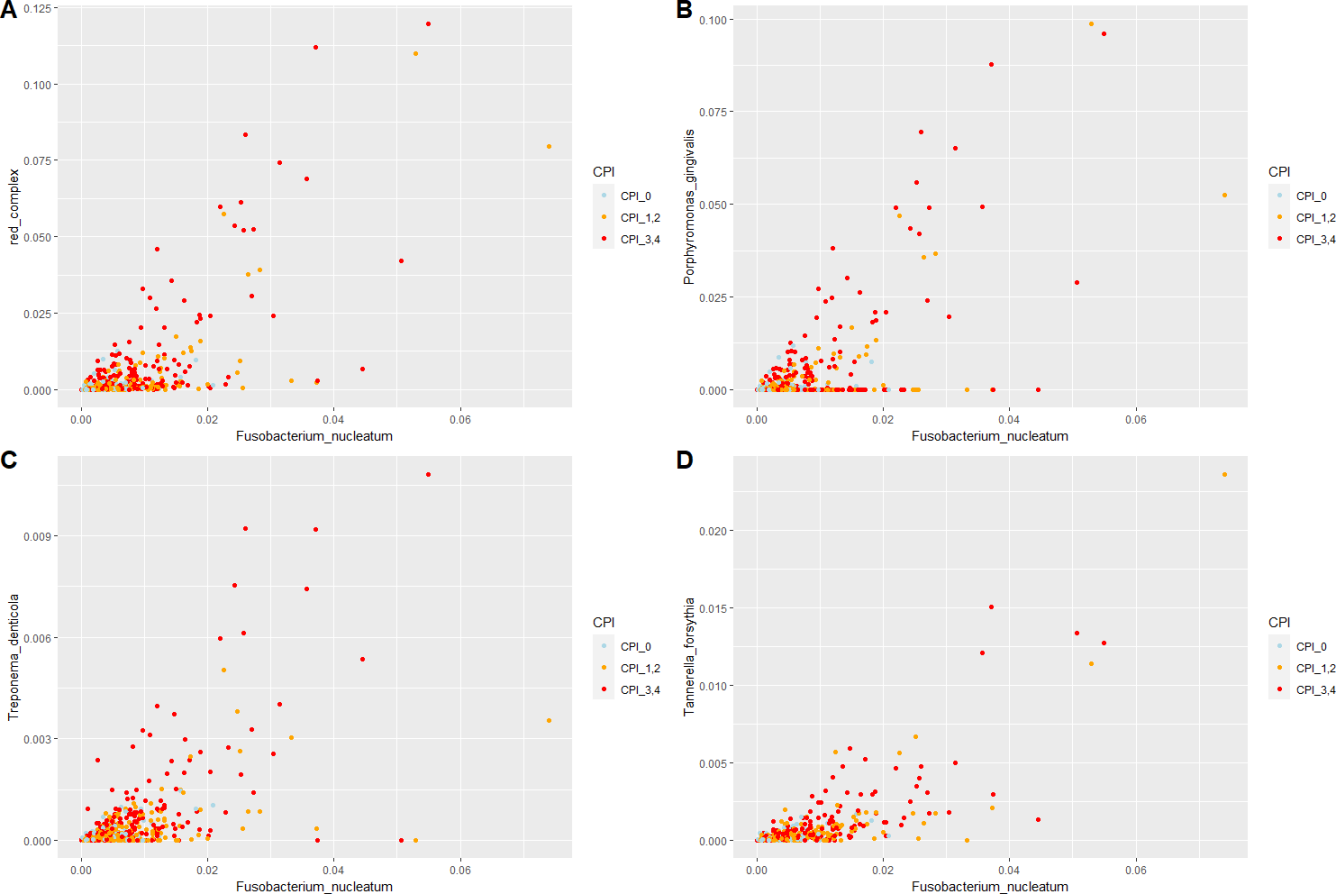
Scatter plots of the relative abundance of *F. nucleatum* and (**A**) red complex, (**B**) *P. gingivalis*, (**C**) *T. denticola,* and (**D**) *T. forsythia*.

To evaluate the dependence of the salivary microbiome on the relative abundance of *F. nucleatum*, bacterial correlations with *F. nucleatum* were analyzed. The red complex and the abundances of *P. gingivalis*, *T. denticola,* and *T. forsythia* (Fig. 3) and *Prevotella intermedia* and *Filifactor alocis* (Table 3) were all significantly correlated with the abundance of *F. nucleatum* (Table 3). Bacterial correlations with each *F. nucleatum* ssp. *animalis*, *nucleatum*, *polymorphum,* and *vincentii* were also analyzed. Bacterial species that correlated with each *F. nucleatum* ssp. differed from those that correlated with the total *F. nucleatum* (Table S2). The abundance of *F. nucleatum* ssp. *vincentii* only significantly correlated with the abundance of all red complex. *F. nucleatum* ssp. was correlated with *P. intermedia,* and *F. nucleatum* ssp. *animalis* and *nucleatum* were significantly correlated with *F. alocis*. The relative abundances of *P. gingivalis*, *T. denticola,* and *T. forsythia* were also correlated with each other (Fig. S2).

**TABLE 3:** Species in saliva with significant correlations with *F. nucleatum^a^*

Species	*r*-value	*P*-value	*q*-value
Bacteroidetes_.G.3. sp._oral_taxon_280	0.5175687	3.63*E*−43	8.24*E*−42
Bacteroidales_.G.2. sp._oral_taxon_274	0.6519596	3.17*E*−75	2.68*E*−73
*Porphyromonas endodontalis*	0.5478822	3.70*E*−49	1.22*E*−47
*Porphyromonas gingivalis*	0.6684861	2.36*E*−80	2.33*E*−78
*Tannerella forsythia*	0.7671538	1.55*E*−119	4.59*E*−117
*Prevotella intermedia*	0.5053445	6.44*E*−41	1.27*E*−39
*Prevotella nigrescens*	0.501717	2.88*E*−40	5.49*E*−39
*Prevotella oris*	0.5737334	8.97*E*−55	4.08*E*−53
Lachnospiraceae_.G.8. sp._oral_taxon_500	0.5167971	5.06*E*−43	1.11*E*−41
Oribacterium sp._oral_taxon_078	0.5166322	5.43*E*−43	1.15*E*−41
Peptococcus sp._oral_taxon_167	0.5216021	6.26*E*−44	1.54*E*−42
*Filifactor alocis*	0.627479	3.41*E*−68	2.01*E*−66
Peptostreptococcaceae_.XI..G.4. sp._oral_taxon_369	0.5354845	1.24*E*−46	3.48*E*−45
Peptostreptococcaceae_.XI..G.5. Eubacterium._saphenum	0.5558212	7.87*E*−51	2.91*E*−49
*Dialister pneumosintes*	0.5204391	1.04*E*−43	2.46*E*−42
*Fusobacterium naviforme*	0.5415454	7.44*E*−48	2.31*E*−46
Fusobacterium sp._oral_taxon_203	0.5692631	9.11*E*−54	3.59*E*−52
Desulfobulbus sp._oral_taxon_041	0.67051	5.28*E*−81	6.24*E*−79
*Campylobacter gracilis*	0.6892243	2.83*E*−87	4.18*E*−85
*Campylobacter showae*	0.5394182	2.01*E*−47	5.93*E*−46
*Treponema denticola*	0.6322753	1.60*E*−69	1.05*E*−67
*Treponema lecithinolyticum*	0.5161551	6.67*E*−43	1.36*E*−41
*Treponema maltophilum*	0.5503057	1.16*E*−49	4.02*E*−48
*Treponema medium*	0.5304105	1.25*E*−45	3.35*E*−44
*Treponema socranskii*	0.5753917	3.76*E*−55	1.85*E*−53
Treponema sp._oral_taxon_237	0.5722737	1.92*E*−54	8.10*E*−53
*Fretibacterium fastidiosum*	0.6045935	3.63*E*−62	1.95*E*−60
Fretibacterium sp._oral_taxon_360	0.641866	3.01*E*−72	2.22*E*−70

^*a*^ Correlation of relative abundance of *F. nucleatum* and species was calculated using the Spearman test, *r* > 0.5, *q* < 0.05. Correlation of the relative abundance of *F. nucleatum* and other salivary species was performed by the Spearman rank test. For these analyses, an adjusted *P*-value of <0.05 was calculated using a Benjamini–Hochberg correction. Salivary species with *r*-value >0.5 and *q*-value <0.05 were shown.

## DISCUSSION

The periodontal status has been associated with the abundance of *F. nucleatum* in saliva, with a recent study showing higher abundance of *F. nucleatum* in saliva in subjects with periodontitis compared with those with periodontal health (15). However, the basis for this association is not clear. In this study, we showed that the relative abundances of *F. nucleatum* in saliva are associated with the periodontal status and that the composition of the salivary microbiota including periodontopathogens is shifted by increasing the relative abundance of *F. nucleatum*. To our knowledge, this is the first evidence for this influence of *F. nucleatum* on the salivary microbiome.

The abundance of red complex has previously been associated with the progression of periodontitis (19), while *F. nucleatum* has been shown to enhance biofilm formation and the level of *F. nucleatum* in subgingival plaque has been associated with the prevalence of periodontal disease. Several studies have reported that the abundance of *F. nucleatum* correlated in saliva and subgingival plaque (11–14, 20). However, the relationship of the abundance of *F. nucleatum* in saliva with the prevalence of periodontal disease and periodontal status has not been widely studied. In our classification of subjects into four groups based on the abundance of *F. nucleatum*, we found a higher rate of subjects with CPI = 3 or 4 (periodontitis) in those with higher levels of *F. nucleatum*. These results suggest that the high relative abundance of *F. nucleatum* leads to the development of periodontal disease and is a risk factor for progression. The finding that the levels of *F. nucleatum* in saliva affect the prevalence of periodontal disease is consistent with the report that the high abundance of *F. nucleatum* in subgingival plaque was detected in patients with periodontitis (4). Ko et al. have shown that the relative abundance of *F. nucleatum* in the saliva of post-periodontal therapy was decreased compared with that of pre-periodontal therapy (21). Therefore, we suggest that the abundance of *F. nucleatum* in saliva might be utilized as a diagnostic marker of periodontal disease. However, these microbiome data are limited data since we had collected saliva samples from a lot of subjects with periodontal health and almost all Japanese people. In the future, we will access the microbiome in more collected samples and expand to other ethnic people.

*F. nucleatum* has been found to co-aggregate with periodontopathogens and to be related to the colonization of these pathogens (22). However, the influence of the relative abundance of *F. nucleatum* on the α- and β-diversity of the saliva microbiome has not been analyzed previously. In the current study, we found that α-diversity was higher in subjects with a high relative abundance of *F. nucleatum*. Higher α-diversity has also been shown in patients with periodontitis (3, 23), and *F. nucleatum* may adhere to various species and become a trigger for the development of periodontal disease. β-Diversity was different between subjects with higher and lower abundance of *F. nucleatum,* which suggests that *F. nucleatum* is associated with the composition of the salivary microbiome and that a change in the abundance of *F. nucleatum* may lead to a change in this composition.

The results for β-diversity indicate that the relative abundance of *F. nucleatum* changes the composition of the salivary microbiome, and it has also been reported that *F. nucleatum* enhances the adhesion and co-aggregation of various oral bacteria (22). Therefore, we analyzed the correlation of the abundance of *F. nucleatum* with the levels of salivary bacteria and found that the abundance of *F. nucleatum* correlated with the total abundance of red complex and that of each red complex component. Fap2/FomA of *F. nucleatum* has been shown to influence these interactions (8, 24), and the current findings are consistent with this report. *T. denticola* co-aggregates with *F. nucleatum* via glycoproteins expressed by *T. denticola* (9), and *T. forsythia* also co-aggregates with *F. nucleatum* (10). These results suggest that the adherence of these red complex bacteria with *F. nucleatum* enhances colonization in saliva, consistent with the finding that the levels of *P. gingivalis*, *T. denticola,* and *T. forsythia* were also correlated with each other.

We also found that the abundance of *F. nucleatum* was correlated with those of *P. intermedia* and *Prevotella nigrescens*. Okuda et al. previously showed that *F. nucleatum* co-aggregated with *P. intermedia* or *P. nigrescens* and that biofilm formation was increased in a co-culture of *F. nucleatum* with *P. intermedia* and *P. nigrescens* (25). Simon-Soro et al. previously showed that oral microbes co-aggregated in human saliva (26). These results suggest that *F. nucleatum* may facilitate the colonization of *P. intermedia* and *P. nigrescens* in saliva or the formation of subgingival plaque involved in *P. intermedia* and *P. nigrescens* and release plaque to saliva. An increased relative abundance of *F. nucleatum* enhances the growth of *F. alocis* in co-culture (27), and *F. alocis* is related to the development and progression of periodontitis as a periodontopathogen (28, 29). Thus, *F. nucleatum* may enhance the colonization of both red complex and *P. intermedia*, *P. nigrescens,* and *F. alocis*. The current study also provides the first evidence of the correlation of *F. nucleatum* with *Fretibacterium* ssp., which Oliveira et al. found to be associated with periodontal disease (30). We also found that bacterial species correlated with the abundance of total *F. nucleatum* is different from those with the abundance of each *F. nucleatum* ssp. An assessment of the microbial community formed between *F. nucleatum* and periodontopathogens including red complex, *Prevotella* ssp., *F. alocis,* and *Fretibacterium* ssp. is required in a future study. The salivary microbiota may undergo dysbiosis with an increase in the abundance of *F. nucleatum,* and this may lead to the progression of periodontal disease, based on the correlation of the relative abundance of *F. nucleatum* with periodontopathogens. However, the level of *F. nucleatum* had a low correlation with that of early colonizers in this study (data not shown) although *F. nucleatum* has been shown to co-aggregate with *Streptococcus gordonii* (31). Our results suggest that the abundance of *F. nucleatum* has no effect on the abundance of *S. gordonii* in saliva. Although 16S rRNA sequencing is difficult to obtain at the species level, we analyzed the salivary microbiome at the species level in order to investigate the abundance of *F. nucleatum*. Shimomura et al. identified the strain level of *F. nucleatum* by using the CRISPR-Cas region (32). In future studies, we will analyze the association abundance of *F. nucleatum* with those of salivary microbes using the above method using the CRISPR-Cas and real-time PCR.

The results show the influence of the abundance of *F. nucleatum* in saliva on periodontal status and the composition of the salivary microbiome, including the red complex and periodontopathogens. The increase in abundance of *F. nucleatum* may change the composition of the oral microbiome and lead to dysbiosis in the oral microbiome. However, the increase in abundance of *F. nucleatum* changing the composition of the oral microbiome was not clear. Future studies will be required to investigate whether changing the abundance of *F. nucleatum* affects the composition of the oral microbiome in longitudinal studies and interventional studies.

## Data Availability

The datasetsdata sets generated and/or analyzed during the current study are available in the Sequence Read Archive (SRA) of the National Centre for Biotechnology Information (NCBI) under the bioproject number PRJNA913920 (Search: PRJNA913920 - NLM (nih.gov)) .

## SUPPLEMENTAL MATERIAL

Supplemental material spectrum.00855-24-s0001.docx Tables S1 and S2; Fig. S1 and S2.

ASM does not own the copyrights to Supplemental Material that may be linked to, or accessed through, an article. The authors have granted ASM a non-exclusive, world-wide license to publish the Supplemental Material files. Please contact the corresponding author directly for reuse.
