# Supplementary material for: Association of Fusobacterium nucleatum in human saliva with periodontal status and composition of the salivary microbiome including periodontopathogens
Source: Microbiol Spectr. 2024 Oct 22;12(12):e00855-24. doi: 10.1128/spectrum.00855-24 (PMC11619574; doi:10.1128/spectrum.00855-24)
Supplement: Supplemental material — Tables S1 and S2; Fig. S1 and S2. [file spectrum.00855-24-s0001.docx]

**Association of Fusobacterium nucleatum in human saliva with periodontal status and composition of the salivary microbiome including periodontopathogens**

Takanori Akase^#^, Junya Inubushi, Yoshiko Hayashi-Okada, Yasumitsu Shimizu^#^

Oral Care R&D Department, Sunstar Inc., Osaka, Japan

**Supplementary Material**

**Supplementary Table S1.** Distribution of CPI in groups classified based on the relative abundance of *F. nucleatum* ssp. (A) *animalis,* (B) *nucleatum,* (C) *polymorphum* and (D) *vincentii. Comparison of distribution of gender, age and CPI between lowest quantile group (Q1) and highest quantile group (Q4) was performed by Fisher exact test.*

(A) *F. nucleatum* ssp. *animalis*

| Parameter | Number of subjects | | | | p-value |
| --- | --- | --- | --- | --- | --- |
|  | Quantile 1 | Quantile 2 | Quantile 3 | Quantile 4 | Q1 vs Q4 |
| Gender  Male Female | 112 (73.2%)  41 (26.8%) | 102 (66.7%)  51 (33.3%) | 111 (73.0%)  41 (27.0%) | 110(71.9%)  43(28.1%) | *p* = 0.898 |
| Age group  20-29  30-39  40-49  50-59  ≥60 | 15 (9.8%)  26 (17.0%)  53 (34.6%)  43 (28.1%)  16 (10.5%) | 17 (11.1%)  40 (26.1%)  48 (31.4%)  36 (23.5%)  12 (7.8%) | 14 (9.2%)  26 (17.1%)  58 (38.2%)  46 (30.3%)  8 (5.3%) | 17 (11.1%)  22 (14.4%)  55 (35.9%)  48 (31.4%)  11 (7.2%) | *p* = 0.797 |
| CPI  0  1, 2  3, 4 | 55 (35.9%)  59 (38.6%)  39 (25.5%) | 30 (19.6%)  64 (41.8%)  59 (38.6%) | 31 (20.4%)  71 (46.7%)  50 (32.9%) | 18 (11.8%)  65 (42.5%)  70 (45.8%) | *p* < 0.001 |

(B) *F. nucleatum* ssp. *nucleatum*

| Parameter | Number of subjects | | | | p-value |
| --- | --- | --- | --- | --- | --- |
|  | Quantile 1 | Quantile 2 | Quantile 3 | Quantile 4 | Q1 vs Q4 |
| Gender  Male  Female | 100 (65.8%)  52 (34.2%) | 111 (72.5%)  42 (27.5%) | 111 (72.5%)  42 (27.5%) | 113 (73.9%)  40 (26.1%) | *p* = 0.136 |
| Age group  20-29  30-39  40-49  50-59  ≥60 | 17 (11.2%)  41 (27.0%)  43 (28.3%)  38 (25.0%)  13 (8.6%) | 18 (11.8%)  30 (19.6%)  57 (37.3%)  38 (24.8%)  10 (6.5%) | 15 (9.8%)  24 (15.7%)  61 (39.9%)  42 (27.5%)  11 (7.2%) | 13 (8.5%)  19 (12.4%)  53 (34.6%)  55 (35.9%)  13 (8.5%) | *p <* 0.05 |
| CPI  0  1, 2  3, 4 | 44 (28.9%)  59 (38.8%)  49 (32.2%) | 38 (24.8%)  74 (48.4%)  41 (26.8%) | 30 (19.6%)  64 (41.8%)  59 (38.6%) | 22 (14.4%)  62 (40.5%)  69 (45.1%) | *p* < 0.005 |

(C) *F. nucleatum* ssp. *polymorphum*

| Parameter | Number of subjects | | | | p-value |
| --- | --- | --- | --- | --- | --- |
|  | Quantile 1 | Quantile 2 | Quantile 3 | Quantile 4 | Q1 vs Q4 |
| Gender  Male Female | 100 (65.4%)  53 (34.6%) | 107 (70.4%)  45 (29.6%) | 115 (75.2%)  38 (24.8%) | 113 (73.9%)  40 (26.1%) | *p* = 0.136 |
| Age group  20-29  30-39  40-49  50-59  ≥60 | 17 (11.1%)  34 (22.2%)  56 (36.6%)  30 (19.6%)  16 (10.5%) | 17 (11.2%)  31 (20.4%)  51 (33.6%)  43 (28.3%)  10 (6.6%) | 10 (6.5%)  25 (16.3%)  60 (39.2%)  48 (31.4%)  10 (6.5%) | 19 (12.4%)  24 (15.7%)  47 (30.7%)  52 (34.0%)  11 (7.2%) | *p* = 0.050 |
| CPI  0  1, 2  3, 4 | 51 (33.3%)  61 (39.9%)  41 (26.8%) | 36 (23.7%)  61 (40.1%)  55 (36.2%) | 20 (13.1%)  71 (46.4%)  62 (40.5%) | 27 (17.6%)  66 (43.1%)  60 (39.2%) | *p <* 0.005 |

(D) *F. nucleatum* ssp. *vincentii*

| Parameter | Number of subjects | | | | p-value |
| --- | --- | --- | --- | --- | --- |
|  | Quantile 1 | Quantile 2 | Quantile 3 | Quantile 4 | Q1 vs Q4 |
| Gender  Male Female | 100 (65.4%)  53 (34.6%) | 112 (73.2%)  41 (26.8%) | 111 (73.0%)  41 (27.0%) | 112 (73.2%)  41 (26.8%) | *p* = 0.173 |
| Age group  20-29  30-39  40-49  50-59  ≥60 | 28 (18.3%)  34 (22.2%)  52 (34.0%)  27 (17.6%)  12 (7.8%) | 12 (7.8%)  33 (21.6%)8  56 (36.6%)  42 (27.5%)  10 (6.5%) | 13 (8.6%)  29 (19.1%)  49 (32.2%)  47 (30.9%)  14 (9.2%) | 10 (6.5%)  18 (11.8%)  57 (37.3%)  57 (37.3%)  11 (7.2%) | *p <* 0.001 |
| CPI  0  1, 2  3, 4 | 53 (34.6%)  61 (39.9%)  39 (25.5%) | 33 (21.6%)  79 (51.6%)  41 (26.8%) | 35 (23.0%)  62 (40.8%)  55 (36.2%) | 13 (8.5%)  57 (37.3%)  83 (54.2%) | *p <* 0.001 |

**Supplementary Table S2.** Species in saliva with significant correlations with *F. nucleatum* ssp. (A) *animalis,* (B) *nucleatum,* (C) *polymorphum* and (D) *vincentii.* Correlation of the relative abundance of *F. nucleatum* spp. and other salivary species was performed by Spearman rank test. For these analyses, an adjusted p-value of <0.05 was calculated using a Benjamini-Hochberg correction. Salivary species with r value > 0.5 and q value < 0.05 were shown.

(A) *F. nucleatum* ssp. *animalis*

| Species | r_value | p_value | q_value |
| --- | --- | --- | --- |
| Actinomycess__sp._oral_taxon_525 | 0.679725 | 4.93E-84 | 3.64E-82 |
| Bacteroidetes_.G.5. sp._oral_taxon_511 | 0.561934 | 3.78E-52 | 1.60E-50 |
| Bacteroidales_.G.2. sp._oral_taxon_274 | 0.651336 | 4.88E-75 | 2.62E-73 |
| Tannerella forsythia | 0.678963 | 8.87E-84 | 5.82E-82 |
| Johnsonella ignava | 0.753151 | 7.42E-113 | 2.19E-110 |
| Peptococcus sp._oral_taxon_167 | 0.569357 | 8.68E-54 | 3.95E-52 |
| Filifactor alocis | 0.522998 | 3.39E-44 | 1.06E-42 |
| Peptostreptococcaceae_.XI..G.5. Eubacterium._saphenum | 0.554819 | 1.29E-50 | 4.47E-49 |
| Peptostreptococcaceae_.XI..G.7. Eubacterium._yurii_subsp._schtitka | 0.697119 | 4.57E-90 | 4.50E-88 |
| Leptotrichia sp._oral_taxon_223 | 0.542235 | 5.38E-48 | 1.77E-46 |
| Desulfobulbus sp._oral_taxon_041 | 0.692416 | 2.16E-88 | 1.82E-86 |
| Campylobacter gracilis | 0.67858 | 1.19E-83 | 7.03E-82 |
| Aggregatibacter actinomycetemcomitans | 0.708489 | 2.99E-94 | 4.42E-92 |
| Treponema lecithinolyticum | 0.56074 | 6.87E-52 | 2.71E-50 |
| Treponema socranskii | 0.511205 | 5.52E-42 | 1.63E-40 |
| Treponema sp._oral_taxon_237 | 0.557836 | 2.91E-51 | 1.08E-49 |
| Treponema sp._oral_taxon_517 | 0.714094 | 2.18E-96 | 4.30E-94 |
| Fretibacterium sp._oral_taxon_358 | 0.627779 | 2.82E-68 | 1.39E-66 |
| Fretibacterium sp._oral_taxon_360 | 0.704633 | 8.27E-93 | 9.78E-91 |

(B) *F. nucleatum* ssp. *nucleatum*

| Species | r_value | p_value | q_value |
| --- | --- | --- | --- |
| Bacteroidales_.G.2. sp._oral_taxon_274 | 0.519194 | 1.79E-43 | 1.51E-41 |
| Tannerella forsythia | 0.54488 | 1.55E-48 | 3.05E-46 |
| Lactobacillus ultunensis | 0.533889 | 2.57E-46 | 3.79E-44 |
| Filifactor alocis | 0.559626 | 1.20E-51 | 3.54E-49 |
| Treponema denticola | 0.523762 | 2.42E-44 | 2.39E-42 |
| Treponema parvum | 0.509248 | 1.26E-41 | 6.77E-40 |
| Treponema sp._oral_taxon_237 | 0.517832 | 3.23E-43 | 2.39E-41 |
| Treponema sp._oral_taxon_253 | 0.513456 | 2.12E-42 | 1.25E-40 |
| Treponema sp._oral_taxon_257 | 0.528289 | 3.23E-45 | 3.82E-43 |
| Treponema sp._oral_taxon_258 | 0.514798 | 1.19E-42 | 7.85E-41 |

(C) *F. nucleatum* ssp. *polymorphum*

| Species | r_value | p_value | q_value |
| --- | --- | --- | --- |
| Prevotella sp._oral_taxon_472 | 0.533281 | 3.39E-46 | 6.67E-44 |
| Selenomonas noxia | 0.503005 | 1.70E-40 | 2.51E-38 |
| Selenomonas sp._oral_taxon_892 | 0.53526 | 1.37E-46 | 4.05E-44 |

(D) *F. nucleatum* ssp. *vincentii*

| Species | r_value | p_value | q_value |
| --- | --- | --- | --- |
| Bacteroidetes_.G.3. sp._oral_taxon_280 | 0.514283 | 1.49E-42 | 8.80E-41 |
| Porphyromonas endodontalis | 0.540293 | 1.34E-47 | 9.87E-46 |
| Porphyromonas gingivalis | 0.670158 | 6.86E-81 | 2.03E-78 |
| Tannerella forsythia | 0.607004 | 8.88E-63 | 1.31E-60 |
| Prevotella oris | 0.577669 | 1.13E-55 | 1.11E-53 |
| Peptostreptococcaceae_.XI..G.4. sp._oral_taxon_369 | 0.510581 | 7.19E-42 | 3.86E-40 |
| Dialister pneumosintes | 0.575122 | 4.33E-55 | 3.66E-53 |
| Fusobacterium sp._oral_taxon_205 | 0.515424 | 9.14E-43 | 6.00E-41 |
| Desulfobulbus sp._oral_taxon_041 | 0.501784 | 2.80E-40 | 1.27E-38 |
| Campylobacter gracilis | 0.503509 | 1.38E-40 | 6.79E-39 |
| Treponema denticola | 0.610267 | 1.29E-63 | 2.55E-61 |
| Fretibacterium fastidiosum | 0.581981 | 1.13E-56 | 1.34E-54 |


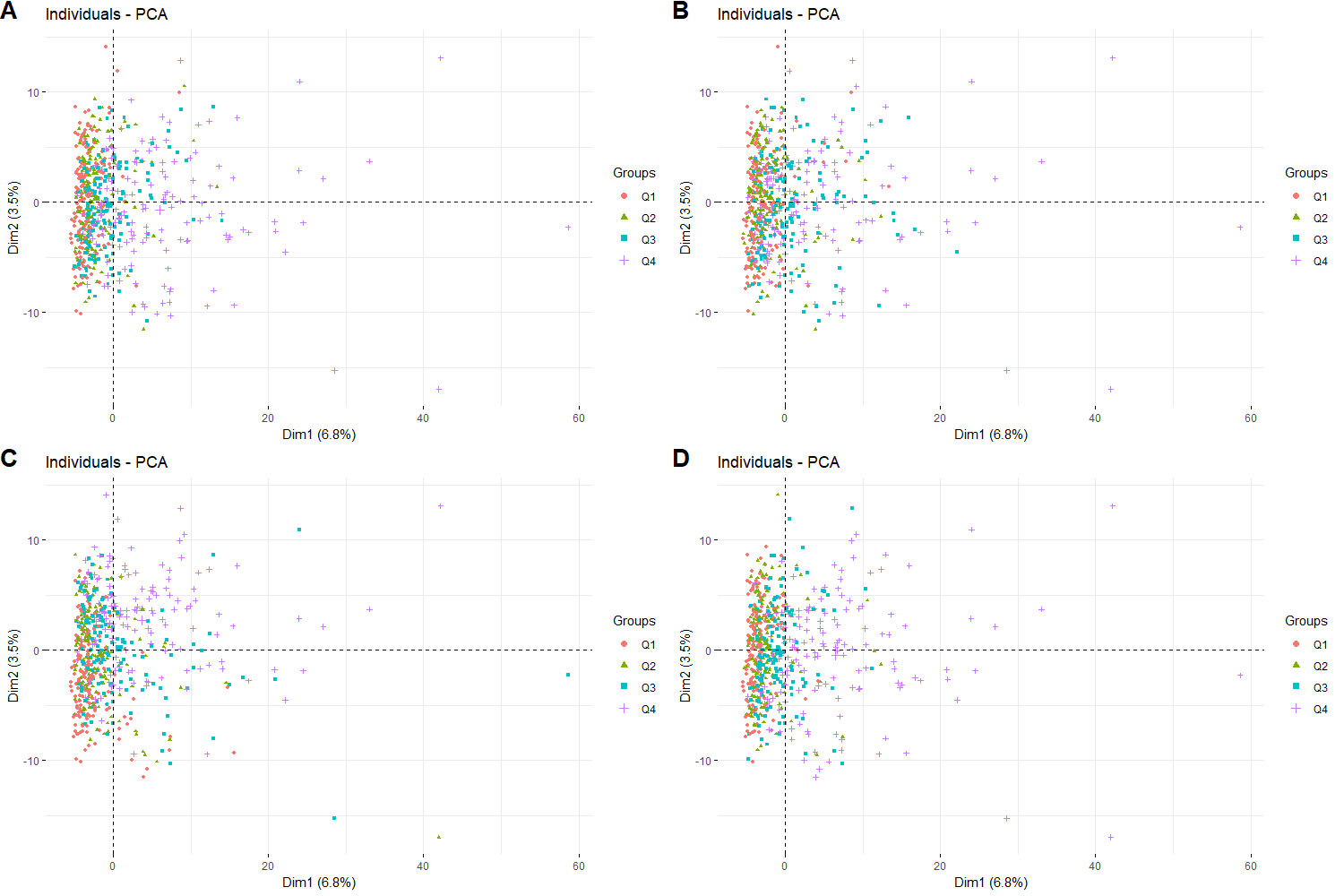
 **Supplementary Fig. S1.** β-diversity of the salivary microbiome classified based on the relative abundance of each *F. nucleatum* ssp. (A) *animalis,* (B) *nucleatum,* (C) *polymorphum* and (D) *vincentii.* Subjects were categorized into four groups based on quantiles of relative abundance of each *F. nucleatum* ssp. Q1: Quantile 1, Q2: Quantile 2, Q3: Quantile 3, Q4: Quantile 4. Principal component analysis of the salivary microbiota was shown. Each point represents an individual sample. Comparison of difference among all groups in PCA was performed by permutational multivariate analysis of variance (PERMANOVA) with a permutation of 999.


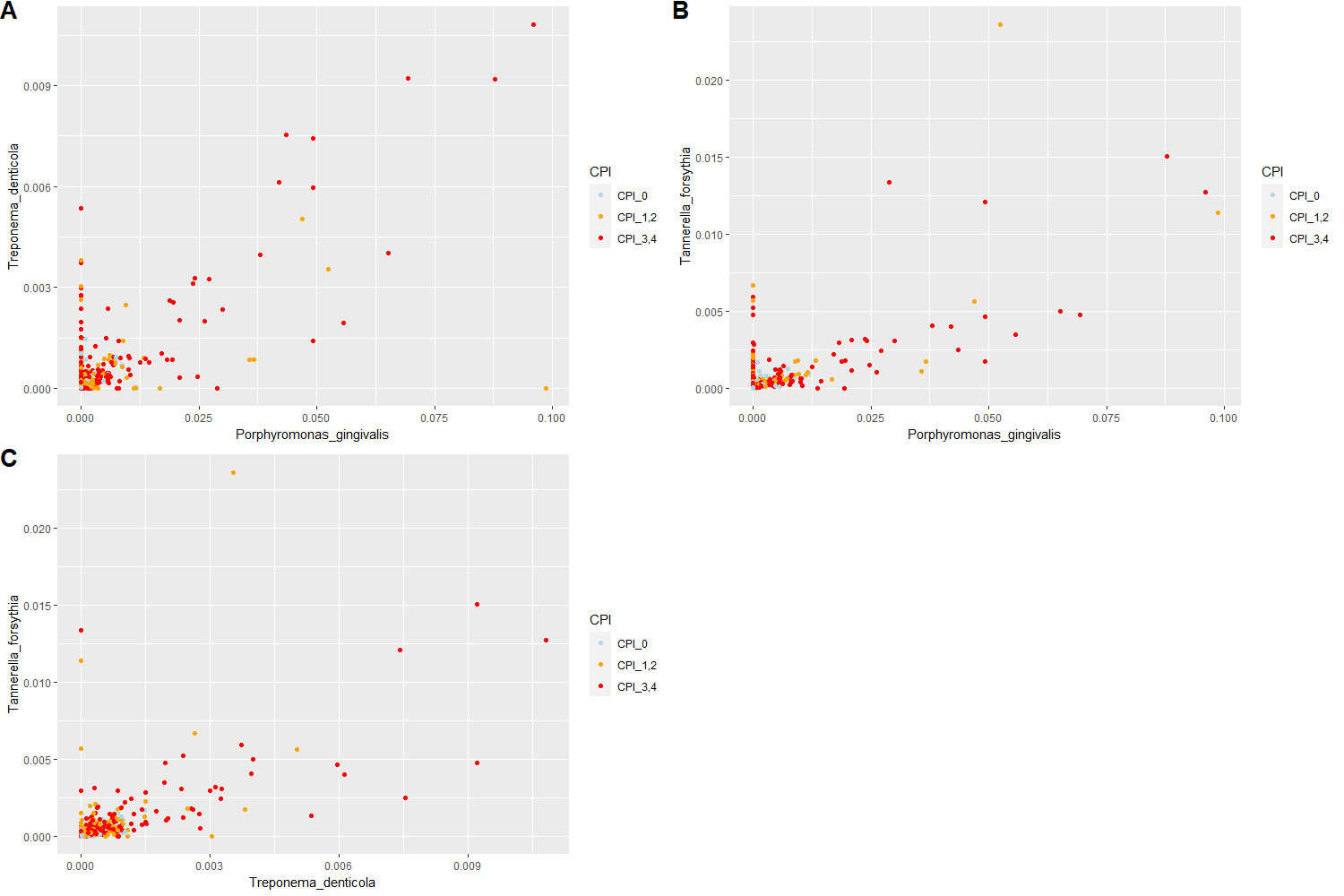


**Supplementary Fig. S2.** Scatter plots of the relative abundance of *P. gingivalis, T. denticola* and *T. forsythia.*
